# A novel *TFG* c.793C>G mutation in a Chinese pedigree with Charcot‐Marie‐Tooth disease 2

**DOI:** 10.1002/brb3.1724

**Published:** 2020-07-14

**Authors:** Ding‐Wen Wu, Yanfang Li, Xinzhen Yin, Baorong Zhang

**Affiliations:** ^1^ Department of Genetics and Metabolism, Children’s Hospital Zhejiang University School of Medicine Hangzhou China; ^2^ Key Laboratory of Diagnosis and Treatment of Neonatal Diseases of Zhejiang Province Hangzhou China; ^3^ Department of Pediatrics, Second Affiliated Hospital Zhejiang University School of Medicine Hangzhou China; ^4^ Department of Neurology, Second Affiliated Hospital Zhejiang University School of Medicine Hangzhou China

**Keywords:** Charcot‐Marie‐Tooth disease 2, distal muscle atrophy, motor nerve conduction velocity, novel mutation, *TFG*

## Abstract

**Introduction:**

Mutations within *TFG* gene were recently reported to cause Charcot‐Marie‐Tooth disease 2 (CMT2). However, only few pedigrees were documented so far. Here, we reported a Chinese CMT2 pedigree with 8 affected cases and a novel *TFG* mutation.

**Methods:**

Clinical evaluation and electrophysiological study were performed in all the affected individuals. Whole‐exome sequencing was conducted, followed by the Sanger sequencing and co‐segregation analysis to verify the variants.

**Results:**

All cases presented with a phenotype of CMT2, including slowly progressive symmetrical muscle atrophy and weakness predominantly in the distal limbs. Sensory loss in the distal limbs was present in the proband and his father. Age at onset ranged from 37 to 44 years, and was younger in male cases, compared with female cases. Nerve conduction study revealed normal motor nerve conduction velocity but decreased compound muscle action potential. Electromyography test revealed fibrillation potential and positive sharp waves. The creatine kinase activity was increased in all cases. After genetic investigations, we identified a novel *TFG* c.793C>G (p.Pro265Ala) mutation in the family. This mutation alters the conserved amino acid residue and is absent in 1000G, ExAC, dbSNP, EP6500, and 200 in‐house controls. It co‐segregated with the disease in the family.

**Conclusions:**

Our report provided additional evidence that the heterozygous *TFG* mutations were associated with CMT2.

## Graphical abstract

TFG was a rare causative gene of CMT. In this study, we reported a novel TFG mutation in a Chinese pedigree with 8 CMT2 patients. Our report provided additional evidence that the heterozygous TFG mutations were associated with CMT2. b1: muscle atrophy of upper limbs in the proband (III‐14) b2: muscle atrophy of lower limbs in the proband (III‐14) b3: muscle atrophy of upper limbs in II‐6 b4: muscle atrophy of lower limbs in II‐6 b5: muscle atrophy of upper limbs in III‐10 b6: muscle atrophy of lower limbs in III‐10 b7: muscle atrophy of upper limbs in III‐8 b8: muscle atrophy of lower limbs in III‐8

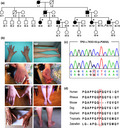

## 1 INTRODUCTION

Charcot‐Marie‐Tooth disease (CMT) is the most common form of inherited neuropathies with great clinical and genetic heterogeneity (Rossor, Polke, Houlden, & Reilly, 2013). The prevalence of CMT is estimated at 17‐40/100000 (Barreto, Oliveira, & Nunes, 2016). According to the nerve conduction velocity (NCV) of median motor, CMT is divided into three types: demyelinating (CMT1) with NCV < 35 m/s, axonal (CMT2) with NCV > 45 m/s, and intermediate CMT (ICMT) with NCV 35–45 m/s (Mathis, Goizet, & Tazir, 2015; Thomas, Guergueltcheva, & Gondim, 2016). Patients with CMT usually suffer from slowly progressive muscle weakness and atrophy of distal extremities and sensory loss of distal end. The symptoms usually begin in the first decade to the third decade but can be great variable. The inherited pattern of CMT includes autosomal dominant, autosomal recessive, and X‐linked. To date, more than 80 genes have been associated with CMT (Pareyson, Saveri, & Pisciotta, 2017). Among these identified genes, *PMP22* duplication is the most common cause, accounting for 40%–50% of all CMT and about 70% CMT1 (Li, Parker, Martyn, Natarajan, & Guo, 2013; van Paassen et al., 2014). The second most common cause of CMT is *GJB1* mutations, which are transmitted with the X‐linked model (Murphy, Laura, & Fawcett, 2012). In the CMT2 subtype, at least 17 genes have been identified, and mutations in *MFN2*, *MPZ*, and *NEFL* are more common than others (Boerkoel, Takashima, & Garcia, 2002). However, the genetic causes of about 60% CMT2 patients remain elusive.

In 2014, mutations in tropomyosin‐receptor kinase fused gene (*TFG*) was identified as a cause of CMT2 (Tsai, Huang, & Guo, 2014). A heterozygous *TFG* c.806G>T (p.Gly269Val) mutation was detected in a large autosomal dominant CMT pedigree with 16 affected individuals and 11 unaffected members. The clinical severity varied from asymptomatic to dependent on the assistance of walkers for ambulation. Functional study showed that *TFG* p.Gly269Val mutation increased the propensity of TFG proteins to form aggregates, resulting in sequestration of mutant and wild TFG protein and thus compromising the protein secretory process. Recently, Khani et al. found *TFG* p.Gly269Val mutation in an Iranian pedigree with CMT2 (Khani, Taheri, & Shamshiri, 2019). Of note, only few CMT2 pedigrees with *TFG* mutations were documented so far. In this study, we reported a four‐generation Chinese CMT2 family with 8 affected individuals and a novel *TFG* c.793C>G mutation. Detailed clinical features were presented below.

## 2 METHODS

### 2.1 Subjects

This study was approved by the Ethics Committees of Second Affiliated Hospital of Zhejiang University School of Medicine. Written informed consents were obtained by all participants. Clinical evaluation was performed by at least two senior neurologists. A total of 8 patients were involved in this family. Nerve conduction study and electromyography (EMG) were performed in available individuals*. PMP22* duplication/deletion was excluded before the whole‐exome sequencing (WES). Besides, 200 normal individuals with no history of neurological disorders were collected as controls.

### 2.2 Genetic investigations

Genomic DNA was extracted from peripheral blood, using QIAamp genomic DNA kits (Qiagen). WES was performed in the proband, followed by the Sanger sequencing to verify the variant. Co‐segregation analysis was conducted in all available familial members. Minor allele frequency (MAF) was searched on ExAC, dbSNP, and 1,000 Genomes Browser. In silico algorithms SIFT, PolyPhen‐2, Mutation Taster, and CADD were used for functional prediction analysis. Variants were classified according to American College of Medical Genetics and Genomics (ACMG) guideline.

## 3 RESULTS

### 3.1 Clinical features

**FIGURE 1:**
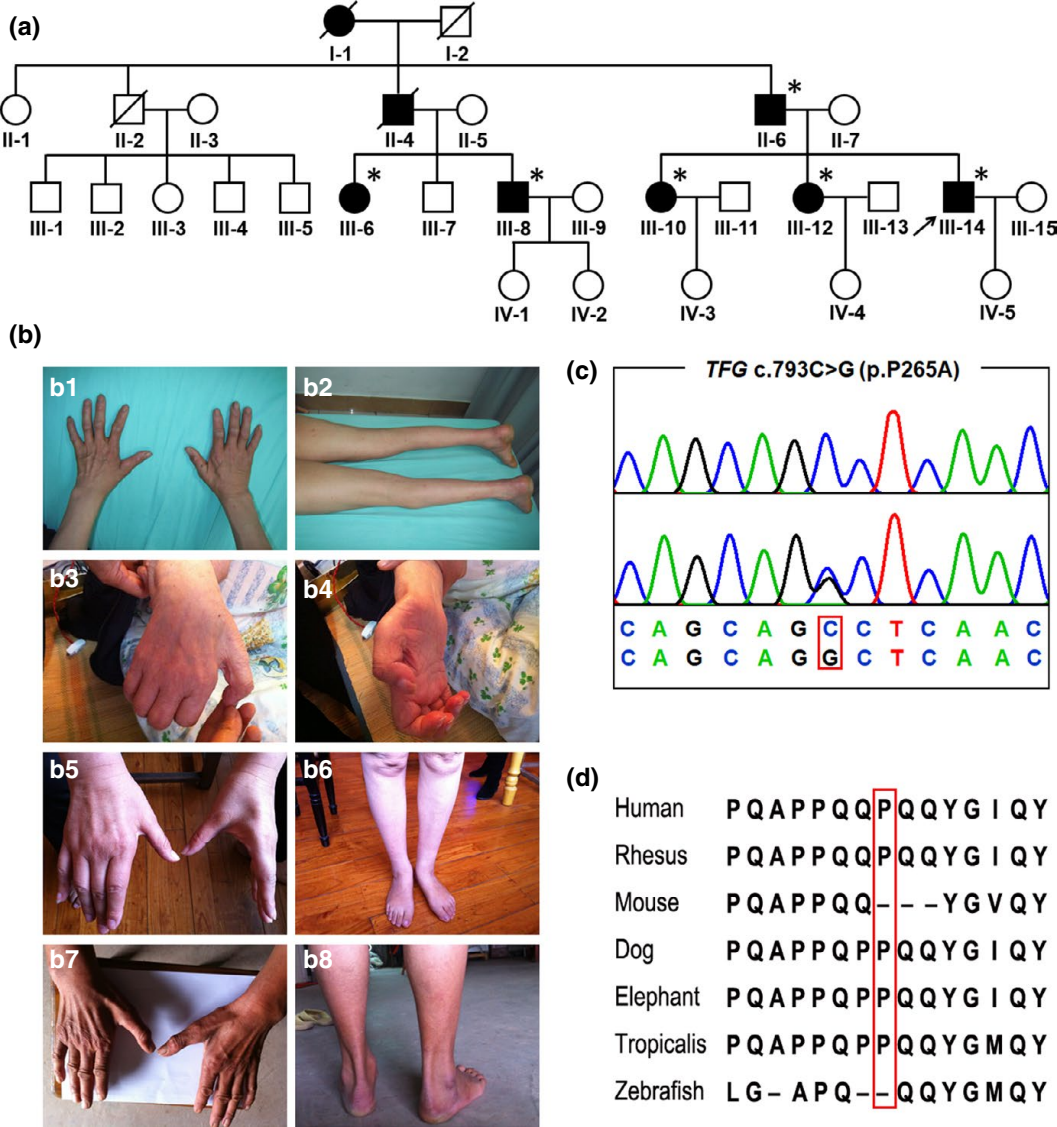
Pedigree, clinical features, and chromatogram of novel *TFG* mutation. (a) Pedigree chart of *TFG* c.793C>G mutation. Squares indicate males; circles indicate females; the black symbols indicate affected individuals; arrows indicate the probands; and asterisks indicate the individual with mutation. (b) Peroneal atrophy and interosseous atrophy were seen in the proband and affected family members. (c) Chromatogram of *TFG* c.793C>G mutation. The upper panel in chromatogram depicts the reference sequence. The lower panel represents heterozygous mutated sequence. (d) The *TFG* p.Pro265Ala mutation resides in an evolutionarily conserved region b1: muscle atrophy of upper limbs in the proband (III‐14) b2: muscle atrophy of lower limbs in the proband (III‐14) b3: muscle atrophy of upper limbs in II‐6 b4: muscle atrophy of lower limbs in II‐6 b5: muscle atrophy of upper limbs in III‐10 b6: muscle atrophy of lower limbs in III‐10 b7: muscle atrophy of upper limbs in III‐8 b8: muscle atrophy of lower limbs in III‐8

A total of 8 affected individuals were included in this four‐generation pedigree (Figure 1a). The inheritance pattern was autosomal dominant. Age at onset ranged from 37 to 44 years. All affected individuals presented with CMT2 phenotypes, including slowly progressive symmetrical muscle atrophy and weakness predominantly in the distal limbs. Sensory loss in the distal limbs was present in the proband and his father. No sensory impairment was detected in other affected individuals. The median motor NCV ranged from 54.6 to 62.1 m/s (Table 1). Compound muscle action potential (cMAP) was decreased. EMG test revealed fibrillation potential and positive sharp waves in the proband and his older sister (III‐12). The creatine kinase (CK) was increased in all cases.

**Table 1:** Clinical manifestations of the affected individuals carrying the *TFG* p.Pro265Ala mutation

	II‐6	III‐6	III‐8	III‐10	III‐12	III‐14
Gender	M	F	M	F	F	M
Age at onset	38	44	37	43	41	38
Age at study	76	49	45	51	48	44
Strength, thumb abduction (MRC)	1	4	3	3	4	4
Strength, foot dorsiflexion (MRC)	0	5	4	4	5	3
Distal muscle atrophy UL	Marked	Mild	Marked	Marked	Mild	Marked
Distal muscle atrophy LL	Marked	Mild	Marked	Moderate	Normal	Marked
Sensory loss	Distal	Normal	Normal	Normal	Normal	Normal
Knee reflex	Areflexia	+	++	++	++	Areflexia
Ankle reflex	Areflexia	Areflexia	Areflexia	Areflexia	Areflexia	Areflexia
Median nerve MNCV (m/s)	NA	54.6	NA	NA	56.5	62.1
Median nerve cMAP (mV)	NA	14.4	NA	NA	11.4	0.922
Peroneal nerve MNCV (m/s)	NA	52.6	NA	NA	42.6	46.4
Peroneal nerve cMAP (mV)	NA	4.3	NA	NA	2.08	2.4
Median nerve SNAP (μV)	NA	52.0	NA	NA	50.4	49.1
Sural nerve SNAP (μV)	NA	26.8	NA	NA	8.9	1.2

Abbreviations: cMAP, compound muscle action potential; LL, lower limbs; MNCV, motor nerve conduction velocity; MRC, Medical Research Council scale; NA, not available; SNAP, sensory nerve action potential; UL, upper limbs.

The proband (III‐14) was a 44‐year‐old man who had a 6‐year history of muscle weakness and atrophy in four extremities. At the age of 38, he felt muscle weakness in his lower limbs after exercise. The weakness slowly progressed in the following months. Half a year after the onset, he noticed muscle atrophy at the distal end of lower limbs. There was no sensory dysfunction at early stage. At the age of 41, he exhibited muscle atrophy in his hands and his fine movement was impaired. In addition, he presented with muscle cramps in his four extremities. Neurological examinations at the age of 44 revealed obvious muscle atrophy in bilateral interosseous muscle and gastrocnemius muscle (Figure 1b, b1‐b2). Fasciculation could be observed in his lower limbs. Muscle strength (Medical Research Council—MRC scale) showed 4/5 for distal upper limbs, 3/5 for distal lower limbs, and 5/5 for proximal limbs. Deep tendon reflexes were absent in the lower limbs and decreased in the upper limbs. Plantar responses were flexor. Cranial examination, cerebellar, and autonomic functions were unremarkable. Tactile and pain sensations were impaired in the distal limbs. Vibratory sensation was normal. Laboratory tests revealed normal blood cell count, liver function, renal function, serum glucose, and low‐density lipoprotein. However, CK was increased, 637 U/L (normal < 171 U/L). The NCV was 62.1 m/s in the median nerve and 46.4 m/s in the peroneal nerve. The cMAP was 0.922 mV in the median nerve and 1.2 μV in the sural nerve. EMG revealed profuse fibrillation potentials and positive sharp waves with long‐duration, high‐amplitude, polyphasic muscle unit action potentials.

The father (II‐6), aged 76 at present, exhibited muscle atrophy at the age of 38. The amyotrophy commenced at the distal end of upper limbs and gradually developed to the lower limbs. Muscle cramps of four extremities were obvious at the early stage of the disease. Also, he complained about pain of fingers in the cold water. At the age of 71 years, he lost ambulation and became bedridden. Neurological examinations revealed extensive muscular atrophy, especially in deltoid, triceps, forearm, interosseous, tibialis anterior, and gastrocnemius muscle (Figure 1b, b3‐b4). Muscle strength revealed 3/5 in the proximal limbs, 1/5 in the distal upper limbs, and 0/5 in the distal lower limbs. Touch and pain sensations were decreased in hands and feet. Vibratory sensation was slightly decreased in ankle joints. Deep tendon reflex was disappeared in all limbs. Plantar responses were flexor. EMG test and laboratory examinations were refused.

The 51‐year‐old sister (III‐10) suffered from muscle atrophy of distal upper limbs at the age of 43. Her amyotrophy was predominant in interosseous, thenar, and gastrocnemius muscle (Figure 1b, b5‐b6). She also complained of recurrent muscle cramps. Neurological examinations revealed muscle strength 3/5 in the distal limbs and 5/5 in the proximal limbs. Knee reflex was normal, while ankle reflex was disappeared. Sensory examinations were negative. Plantar responses were flexor. Another sister (III‐12) had initial symptoms of muscle weakness and cramps in the lower limbs at the age of 38. Several months later, she noticed muscle atrophy in her hands. Neurological examinations revealed muscle atrophy in bilateral interosseous muscle, 4/5 muscle strength in the distal upper limbs. Fasciculation was apparent in her lower limbs. There was no sensory impairment in all limbs. CK was increased, 358 U/L (normal < 171 U/L). The NCV revealed decreased cMAP of peroneal nerve and tibial nerve. EMG revealed fibrillation potentials with high‐amplitude, polyphasic muscle unit action potentials. His 45‐year‐old cousin (III‐8) had an age at onset of 38 years. The initial symptoms included weakness of hands and atrophy of interosseous muscle. Muscle cramps were also complained. Muscle atrophy was seen in interosseous muscle, thenar muscle, and short peroneal (Figure 1b, b7‐b8). Muscle strength was 3/5 in the distal upper limbs and 4/5 in the distal lower limbs. Sensory examination was unremarkable. Another cousin (III‐6), aged 49, exhibited muscle atrophy of limbs at the age of 45. Her muscle strength was 4/5 in the distal upper limbs and 5/5 in remaining muscles. No sensory loss was present in her limbs. CK was 247 U/L (normal < 171 U/L). NCV revealed decreased cMAP of peroneal nerve and tibial nerve.

Individual III‐7 (aged 47) had no symptom of muscle atrophy and weakness. His EMG test was unremarkable, with normal motor NCV (61.1 m/s in the median nerve, 46.0 m/s in the peroneal nerve) and normal cMAP (9.8 mV in the median nerve, 10.1 mV in the peroneal nerve). The SNAP was 10.3 and 17.1 μV in the median nerve and sural nerve, respectively. CK was within the normal level (66 U/L). The other familial members II‐1, III‐1, III‐2, III‐3, III‐4, and III‐5 did not exhibit muscle weakness or atrophy at present. II‐2 had passed away and was reported not to have muscle atrophy. NCV study and EMG test were not performed in these members.

### 3.2 Genetic analysis

Using WES, we identified a heterozygous *TFG* (NM_001195478) mutation c.793C>G in the proband (Figure 1c). This mutation causes a substitution of proline 265 with alanine (p.Pro265Ala) and resides in an evolutionarily conserved region (Figure 1d). This mutation was also detected in II‐6, III‐6, III‐8, III‐10, and III‐12, all of whom were affected individuals. We did not find this mutation in II‐1, III‐1, III‐2, III‐3, III‐4, III‐5, and III‐7. Co‐segregation with the disease was confirmed in the family. This mutation was located in the conserved amino acid residue and was absent in 1000G, ExAC, dbSNP, EP6500, and our 200 in‐house controls. It is predicted to be tolerable by SIFT, benign by PolyPhen‐2, disease causing by Mutation Taster, and damaging by CADD. According to the ACMG guideline, this mutation should be classified as “likely pathogenic.”

## 4 DISCUSSION

Here, we presented a novel *TFG* mutation in a Chinese CMT2 pedigree. The mutation p.Pro265Ala alters the conserved amino acid residue and is absent in public genomic database and our in‐house controls. In addition, the location of this mutation was adjacent to the pathogenic mutation p.Gly269Val. The co‐segregation in the family revealed that this mutation might be causative for the disease. Due to the technical and material limitations, the evaluation of functional alternation of the mutant protein was not conducted. Since loss‐of‐function mechanism has been reported as molecular pathogenesis of TFG, we believed this novel mutation may be biologically pathogenic.

The patients in this pedigree exhibited symmetrical muscle atrophy and weakness predominantly in the distal limbs. Distal sensory impairment was present in the proband and his father. Motor nerve conduction studies were within normal limits. EMG showed denervation and reinnervation. Based on the clinical and electrophysiological findings, these patients met the diagnosis criteria of CMT2. In some patients, sensory impairment was not present. It is possible that their sensory loss was mild, similar to the CMT2 patients with *TFG* p.Gly269Val mutation (Tsai et al., 2014). Interestingly, some patients in our study had onset of muscle atrophy in the upper limbs, which is consistent with the patients described by Fabrizi, Høyer, & Taioli (2020). In addition, four patients in this study had muscle cramps and fasciculations, which were not common in CMT2. Previous studies revealed that *TFG* mutations were associated with hereditary motor and sensory neuropathy with proximal dominant involvement (HMSN‐P), (Alavi, Shamshiri, & Nafissi, 2015; Ishiura, Sako, & Yoshida, 2012) which is characterized by predominantly proximal muscle weakness and atrophy, widespread fasciculations, cramps, and late‐onset distal sensory deficit. It seems that the clinical features of our patients had an overlap between CMT2 and HMSN‐P. Actually, patients carrying *TFG* p.Gly269Val have been reported in patients with either CMT2 or HMSN‐P (Khani, Shamshiri, Alavi, Nafissi, & Elahi, 2016; Khani et al., 2019; Tsai et al., 2014) This implied a continuum of phenotypes in HMSN‐P and CMT patients with *TFG* mutations (Fabrizi et al., 2020; Khani et al., 2019).

Mutations within *TFG* have been associated with several distinct phenotypes, including HMSN‐P, (Alavi et al., 2015; Ishiura et al., 2012) hereditary spastic paraplegia (HSP), (Tariq & Naz, 2017) neuroaxonal dystrophy “plus” syndrome, (Catania, Battini, & Pippucci, 2018) and motor neuron disease with sensory neuropathy (Li, Meng, & Wu, 2019). This implied the clinical heterogeneity in patients with *TFG* mutations. Even the identical *TFG* mutation could cause different phenotypes. Different symptoms were also found across the family members. In addition, the presence of fasciculation and cramps is prominent in HMSN‐P. The different clinical features among patients with *TFG* mutations required further explanation. Other modifying genes or environmental factors are potential contributing causes. Alternatively, age at examination may affect the phenotypic differences.

TFG is ubiquitously expressed in human neurons, including the brain, spinal motor neurons, and dorsal root ganglia (Yagi, Ito, & Suzuki, 2016). Different mutations in TFG cause disparate clinical phenotypes, implying that each mutation has its unique pathogenic mechanism. Alternatively, different expressions of TFG in peripheral axons, lower motor neurons, and upper motor neurons might influence the phenotypes. Although the full scope of TFG functions is still unclear, recent studies revealed that TFG functions at endoplasmic reticulum exit sites and regulates secretory protein vesicle biogenesis and egression from the endoplasmic reticulum (Beetz, Johnson, & Schuh, 2013; Yagi et al., 2016). Therefore, impaired endoplasmic reticulum function might be the major mechanisms of mutant TFG in TFG‐related disorders.

## 5 CONCLUSIONS

In summary, we reported a novel *TFG* c.793C>G mutation in a Chinese pedigree with CMT2. Our results provided additional evidence that heterozygous *TFG* mutations were associated with CMT2.

## CONFLICT OF INTEREST

The authors declare no potential conflicts of interest.

## AUTHOR CONTRIBUTIONS

Ding‐Wen Wu contributed to funding, data acquisition, analysis and interpretation, and drafting of the manuscript. Yanfang Li contributed to data acquisition, analysis and interpretation, drafting of the manuscript. Xinzhen Yin contributed to data acquisition. Baorong Zhang contributed to study design, study supervision, data acquisition, analysis and interpretation of data, and critical revision of the manuscript.

## Data Availability

The data supporting the results of this study are publicly available.
